# *ALPL* Mutations With Dominant‐Negative Effect in Infantile Hypophosphatasia Monozygotic Twins

**DOI:** 10.1155/humu/9913394

**Published:** 2026-04-15

**Authors:** Luna Hao, Na Huang, Yilun Tao, Hui Li, Juyu Zhuang, Xiaoyun Li, Zekun Hao, Feng Zhao

**Affiliations:** ^1^ Department of Pediatrics, Changzhi Maternal and Child Health Care Hospital, Changzhi, Shanxi, China; ^2^ Medical Genetic Center, Changzhi Maternal and Child Health Care Hospital, Changzhi, Shanxi, China, mgz-muenchen.com; ^3^ College of Clinical Medicine for Obstetrics & Gynecology and Pediatrics, Fujian Medical University, Fuzhou, Fujian, China, fjmu.edu.cn; ^4^ Department of Nephrology, Rheumatology and Immunology, Fujian Children′s Hospital, Fuzhou, Fujian, China; ^5^ College of Basic Medicine, Shanxi Medical University, Jinzhong, Shanxi, China, sxmu.edu.cn

## Abstract

**Background and Aims:**

Hypophosphatasia (HPP) is a rare inborn error of metabolism caused by *ALPL* gene mutations, resulting in deficient tissue‐nonspecific alkaline phosphatase (ALP) activity. We investigated genotype–phenotype correlations in a monozygotic female twin pair with infantile HPP.

**Methods:**

Peripheral blood samples were collected from two female twins with HPP and their family members. Genomic DNA was extracted, and variations were detected using whole exome sequencing. Zygosity was confirmed via KING 2.3.1 software. Pathogenic variants were validated using Sanger sequencing, mutation analyses, and bioinformatics.

**Results:**

The twins presented with bulging anterior fontanel at 3 months of age. At 6 months, serum ALP levels decreased, and skeletal dysplasia, hypercalcemia, and nephrocalcinosis developed. One twin died of pneumonia at 11 months; the other remained alive beyond 15 months. Monochorionic diamniotic placentation and a twin pair kinship coefficient (0.4879) confirmed monozygosity. Exome sequencing revealed that the twins carried compound heterozygous *ALPL* mutations c.299C>T (p.Thr100Met) and c.1271T>C (p.Val424Ala). The maternally inherited allele c.1271T>C, which was suspected to be pathogenic according to the American College of Medical Genetics and Genomics (ACMG) guidelines, was reported in a homozygous Chinese adult with HPP. The paternally inherited allele c.299C>T was rated as pathogenic by ACMG. In vitro, *ALPL* c.299C>T‐related mutants exhibited reduced residual ALP activity owing to a dominant‐negative effect. Analysis of c.299C>T‐related mutations in the *ALPL* Gene Variant Database revealed statistically significant differences in a dominant‐negative effect and lower residual ALP activity of allelic mutants in early‐onset versus late‐onset HPP (*p* < 0.05). The dominant‐negative effect correlated positively with residual ALP activity (*r*_*s*_ = 0.889, *p* < 0.05).

**Conclusion:**

Compound heterozygous *ALPL* mutations c.299C>T (p.Thr100Met) and c.1271T>C (p.Val424Ala) were causative factors of infantile HPP in the monozygotic twins, providing insights into how dominant‐negative effects influence HPP severity.

## 1. Introduction

Hypophosphatasia (HPP) is a rare inherited metabolic disorder that is widely recognized as the primary clinical feature of abnormal bone and dental mineralization. Its multisystemic complications include hypercalcemia, decreased parathyroid hormone levels, hypercalciuria with renal calcinosis, vitamin B_6_–responsive seizures, muscle weakness, and growth retardation. HPP is caused by deactivating mutations in the *ALPL* gene (MIM#171760), which encodes tissue‐nonspecific alkaline phosphatase (TNSALP) on the extracellular membrane [1–3]. Historically, HPP has been subdivided into six clinical types by age at onset and the severity of skeletal lesions: perinatal lethal, perinatal benign, infantile (OMIM#241500, onset < 6 months), childhood (OMIM#241510, onset ≥ 6 months), adult (OMIM#146300), and odonto (OMIM#146300) HPP [2, 4–6]. Drawing on data from the Global HPP Registry, a binary classification has been proposed, categorizing patients as early‐ (< 6 months) or late‐onset (≥ 6 months) based on age at first disease manifestation [7]. Nonetheless, certain asymptomatic and healthy *ALPL* variant carriers exhibit the biochemical phenotype [8]. As a single‐gene disorder, HPP has a Mendelian inheritance pattern but exhibits diverse gene expression [9]. Mild HPP mostly exhibits autosomal dominant transmission, moderate HPP is autosomal recessive or autosomal dominant, and severe HPP is predominantly autosomal recessive in perinatal and infantile HPP, with a low prevalence of 1/100,000 in North America and 1/300,000 in Europe [5, 6, 10]. Perinatally lethal HPP is fatal shortly after birth. Furthermore, without enzyme replacement therapy, 50% of infants with early‐onset HPP do not survive beyond 12 months of age [2, 3, 11]. Phenotypic heterogeneity of HPP is associated with a dominant‐negative effect of the *ALPL* gene. Autosomal recessive patients with moderate to severe HPP carry at least one mutant allele with dominant‐negative effects, and mutant residual ALP activity decreases significantly [9, 10, 12]. Although most individuals with familial HPP have similar symptoms, few siblings carry the same *ALPL* mutation with discordant disease severity, implying that genetic and unexplained environmental or epigenetic factors may be involved in the pathogenesis of HPP [2, 3, 5, 9, 13]. Monozygotic twins with concordant gene sequences and similar intrauterine growth environments may serve as ideal models to estimate the phenotypic heritability of monogenic genetic diseases. Herein, we report a case of two monozygotic female twins with infantile HPP admitted to Changzhi Maternal and Child Health Hospital (Shanxi, China). The genotype–phenotype correlations of *ALPL* gene variants were examined to enhance our understanding of the dominant‐negative effect in HPP.

## 2. Materials and Methods

### 2.1. Study Participants

Two 6‐month‐old female twins diagnosed with HPP were admitted to Changzhi Maternal and Child Health Hospital in August 2023, and their family members were enrolled in this study [14–16]. The patients and their family members provided informed consent; for participants aged under 18 years, informed consent was provided by their guardians. This study was approved by the Medical Ethics Committee of Changzhi Maternal and Child Health Hospital (Approval No. CZSFYLL2024‐006).

### 2.2. Clinical Information

Demographic and clinical data were collected, including medical history (present and past illnesses), family health information, birth history, and growth and developmental milestones. Physical examinations included weight, length, head circumference, fontanels, thorax, limbs, and nervous system. Growth Standards for Children Under Age 7 Years (WS/T423‐2022, China) were applied to evaluate growth and development [17].

### 2.3. Laboratory Tests and Imaging

Biochemical evaluation of the affected family included serum ALP, serum pyridoxal 5 ^′^‐phosphate (PLP), serum calcium, serum phosphorus, urine calcium‐to‐creatinine ratio, liver and kidney function markers, 25‐hydroxy vitamin D, and parathyroid hormone. Imaging studies in the patients comprised plain radiographs of the limbs, thorax, and abdomen; cranial computed tomography (CT) and magnetic resonance imaging (MRI); and abdominal ultrasonography and CT.

### 2.4. Twins′ Ovality and Kinship Identification

Ovality and genetic information can be used to validate identical twins. Twin′s ovality was identified based on the number of placentas, chorionic villi, and amniotic membranes at birth and a genome‐wide association study. KING 2.3.1 software (https://www.kingrelatedness.com/) was sufficiently robust to estimate the genetic relationship. The pairwise kinship coefficient ranges included > 0.354, 0.177–0.354, 0.0884–0.177, and 0.0442–0.0884, corresponding to duplicate or monozygotic twins, first‐, second‐, and third‐degree relationships, respectively.

### 2.5. Genetic Testing

Peripheral blood was drawn from the twin infants and their family members. Genomic DNA was extracted from the blood using standard procedures (MyGenostics, Beijing, China). Enrichment libraries were screened for whole exome sequencing (WES) using DNBSEQ‐T7. Whole genome copy number variant (CNV) information was obtained using the CNVkit software (https://cnvkit.readthedocs.io/en/stable/). The variants were annotated using ANNOVAR software (http://annovar.openbioinformatics.org/en/latest/), following the American College of Medical Genetics and Genomics (ACMG) standards and guidelines for the interpretation of genetic variants [18] and association with multiple databases, including 1000 Genomes, ESP6500, dbSNP, EXAC, in‐house (MyGenostics, Beijing, China), and HGMD, for variant pathogenicity prediction. Variants were predicted using SIFT, PolyPhen‐2, MutationTaster, and GERP++. Sanger sequencing and pedigree analysis were used to verify all mutations of interest. Sequencing primers were designed using online Primer 3 software with cDNA sequences (NM_000478.6).

### 2.6. Statistical Analysis

From the Global *ALPL* Gene Variant Database (Johannes Kepler University Linz; https://alplmutationdatabase.jku.at/), data were extracted, including genotype, phenotype, mutant residual ALP activity (percentage), and dominant‐negative effects (percentage). *ALPL* mutation‐related genotypes of this study were classified into two groups based on clinical phenotype: early‐onset (< 6 months), encompassing perinatal and infantile HPP, and late‐onset (≥ 6 months), encompassing childhood, adult, and odonto HPP. For each genotype, the mean values for mutant residual ALP activity (percentage) and dominant‐negative effect (percentage) were derived by averaging the corresponding data from its two alleles, whether variant or wild type. SPSS 24.0 and GraphPad Prism 8.0 software were used for data processing and graphical display. If data were independent, normally, or approximately normally distributed and had equal variance, a comparison between two groups was performed using an independent two‐sample *t*‐test; otherwise, a nonparametric test was used. Two groups of measurement values were analyzed for linear correlations. Pearson′s correlation was used for normal or approximately normal distributions, and Spearman′s correlation was used for nonnormal distributions. A correlation coefficient of 0.8–1.0 indicated a high correlation. A *p* value < 0.05 for a two‐sided test was considered statistically significant.

### 2.7. 3D Modeling

A 3D model was constructed by alignment with the known 3D structure of the human TNSALP (https://www.rcsb.org/structure/7YIX). *ALPL* mutations in this study were visually localized using open‐source PyMOL software (http://www.pymol.org/).

## 3. Results

### 3.1. Clinical Data

The patients were born to a nonconsanguineous family. Their elder brother and parents were healthy, exhibiting no signs or symptoms of bone pain, frequent fractures, muscle weakness, or premature tooth loss. Prenatal ultrasonography and placental examination at delivery confirmed monochorionic, diamniotic twins. Prenatal ultrasonography did not show any bending or decreased echogenicity of the long bones of the twin fetuses. The mother underwent a cesarean section delivery at 37^+5^ weeks of gestation (because of the twin pregnancy and severe preeclampsia) with clear amniotic fluid and the absence of asphyxia or hypoxia. In the neonatal period, the twins simultaneously presented with bulging anterior fontanel at 3 months of age and were hospitalized at 6 months of age. They could laugh but were unable to hold their head upright. They showed no vomiting but had weak feeding. On focused examination, the twins exhibited a consistent cephalic and facial appearance with large and prominent eyes, bulging fontanel, normal cranial hardness, and no thoracic deformities with rib ectropion, flexion and floppiness of the limbs, low muscle tone, Class IV muscle strength, and negative neurologic signs. In case of a low developmental quotient, the assessment of growth and development for age according to the Growth Standards for Children Under Age 7 Years (Table 1) showed that at birth, the older sister (II‐2) had a low weight, but the length and head circumference were in the low‐to‐middle range. At birth, the younger sister (II‐3) had a low‐to‐medium weight, length, and head circumference. At 6 months of age, the weight, length, and head circumference were low for II‐2 and II‐3. Both girls were hospitalized at age 7 months for pneumonia and were discharged after conventional treatment. After discharge, the bulging fontanel remained unchanged, with slow weight gain and no respiratory distress. II‐3 died at home at 11 months of age due to a second episode of pneumonia. At the age of 15 months in the last follow‐up, II‐2 was alive with low weight, length, and head circumference, a slightly unstable elevated head, no sitting ability, and no teeth, and her speech included combined syllables.

**Table 1:** Clinical data of monozygotic twin female infants with hypophosphatasia.

Twins (label)	Older sister (II‐2)	Younger sister (II‐3)
1. Age at first presentation (months)	3	3
2. Perinatal history		
Gestational age (weeks)	37^+5^	37^+5^
Apgar scores (1/5 min)	9/10 (−1 for color)	9/10 (−1 for color)
Birth weight (kg)	2.45^a^	2.70^b^
Length (cm)	47^b^	48^b^
Head circumference (cm)	32.3^b^	32.2^b^

3. First visit		
Age (months)	6	6
Weight (kg)	4.2^a^	4.4^a^
Length (cm)	54^a^	51^a^
Head circumference (cm)	36^a^	35^a^
Growth quotient (DQ)	26	30
Thoracic malformation	No	No
Anterior fontanel bulging	Yes	Yes
Cerebrospinal fluid pressure (30–80 mmH_2_O) ^∗^	65	70
Serum alkaline phosphatase (98–532 U/L) ^∗^	< 10.0	< 10.0
Serum pyridoxal 5 ^′^‐phosphate (3–30 ng/L) ^∗^	46.28	43.04
Serum calcium (2.1–2.8 mmol/L) ^∗^	3.62	3.35
Serum phosphorus (1.6–2.5 mmol/L) ^∗^	1.58	1.65
Serum potassium (4.2–5.9 mmol/L) ^∗^	3.02	2.88
Urinary calcium/creatinine (0–0.2 mg/mg) ^∗^	0.95	2.49
Parathyroid hormone (1.17–8.59 pmol/L) ^∗^	< 0.21	< 0.21
25‐hydroxy vitamin D (20–50 ng/mL) ^∗^	46.28	43.04
Hemoglobin (97–141 g/L) ^∗^	87	79
Serum urea (0.8–5.3 mmol/L) ^∗^	8.61	9.01
Serum creatinine (13–33 *μ*mol/L) ^∗^	44.41	47.07

4. Last follow‐up		
Age (months)	15^c^	11^d^
Weight (kg)	7.0^a^	ND
Length (cm)	72^a^	ND
Head circumference (cm)	42^a^	ND

*Note:* The growth and development indicators are based on the percentile method of nutritional status assessed according to “Growth Standards for Children Under Age 7 Years” (WS/T423‐2022, China) [17].

Abbreviations: DQ, development quotient; ND, no data.

^a^< 3rd percentile, in the low level.

^b^3rd–25th percentile, in the low to middle level.

^c^II‐2 was alive at age 15 months.

^d^II‐3 died of pneumonia at age 11 months.

^∗^Reference range.

### 3.2. Laboratory and Imaging Findings

The serum ALP activity and PLP concentrations were assessed in the patients′ first‐degree relatives. The elder brother′s biochemical profile was unremarkable. However, both parents exhibited the biochemical phenotype: The father had low ALP (32 U/L; reference range: 98–532 U/L) and elevated PLP (51 ng/L; reference range: 3–30 ng/L), as did the mother (ALP: 80 U/L; PLP: 35 ng/L). In the affected twins, the serum ALP activity was too low to be measured. Serum and urine calcium and PLP levels were elevated, while parathyroid hormone was decreased. Serum phosphorus and 25‐hydroxy vitamin D levels were normal. Acute kidney injury (AKI) was present, as evidenced by elevated serum creatinine (1.5× reference value) [19], urea nitrogen, and calcium. Hemoglobin fell below 9.5 g/L (< 5th percentile for age and sex), likely attributable to both AKI and poor feeding. No abnormalities in cerebrospinal fluid pressure, routine biochemistry, or bacterial cultures were detected (Table 1). Radiographs of the twin female infants (II‐2 and II‐3) showed similar findings. Cranial CT revealed mild ventricular dilatation, widened fontanel sutures, cranial hypodensity, slightly weaker cranial bones, and incomplete skull ossification (Figure 1b,c). However, cranial MRI did not reveal any obvious abnormalities (Figure 1d). Renal ultrasonography and CT revealed hyperdense foci in the medullary collecting system of the kidneys, suggestive of renal calcinosis (Figure 1e). Skeletal radiographs suggested that the bones of the limbs were demineralized, and some were curved in the diaphysis. Skeletal radiographs revealed demineralization and diaphyseal curvatures of the limb bones, with hypodense metaphyseal shadows, widened physes, and delayed ossification (Figure 1f,g).

Figure 1Clinical and imaging findings of monozygotic female twin infants (II‐2 and II‐3) with hypophosphatasia. The twins exhibited similar cranial and facial features at presentation (6 months of age); hence, high‐definition representative images from a single twin are shown in (a–g). (a) Photograph of II‐2 showing characteristic head and facial appearance. (b, c) Axial cranial CT images from II‐2. (d) Sagittal cranial MRI from II‐3. (e) Coronal abdominal CT image from II‐3. (f, g) Skeletal survey radiographs from II‐2. CT, computed tomography; MRI, magnetic resonance imaging.

**Figure:**
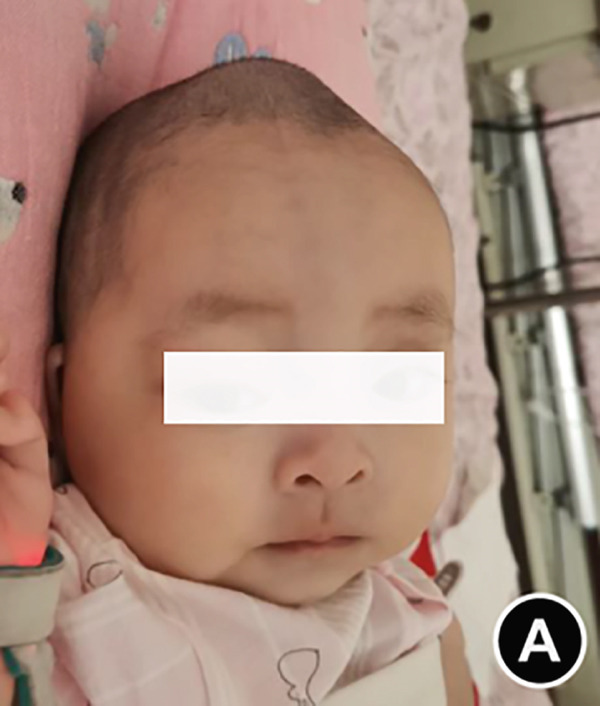
(a)

**Figure:**
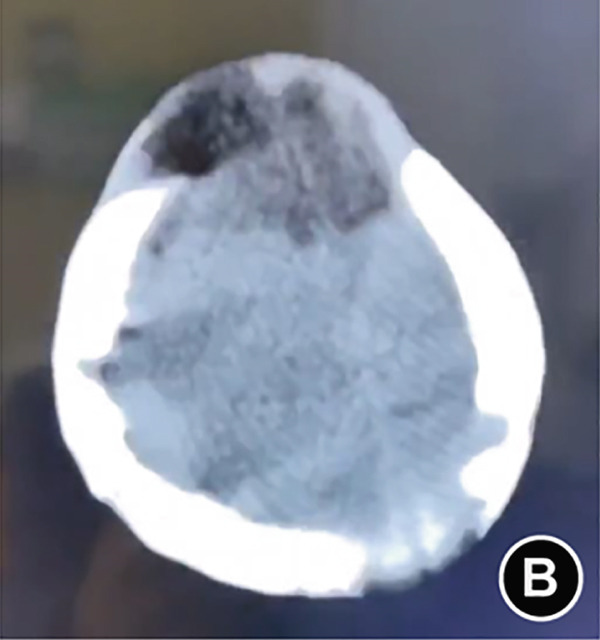
(b)

**Figure:**
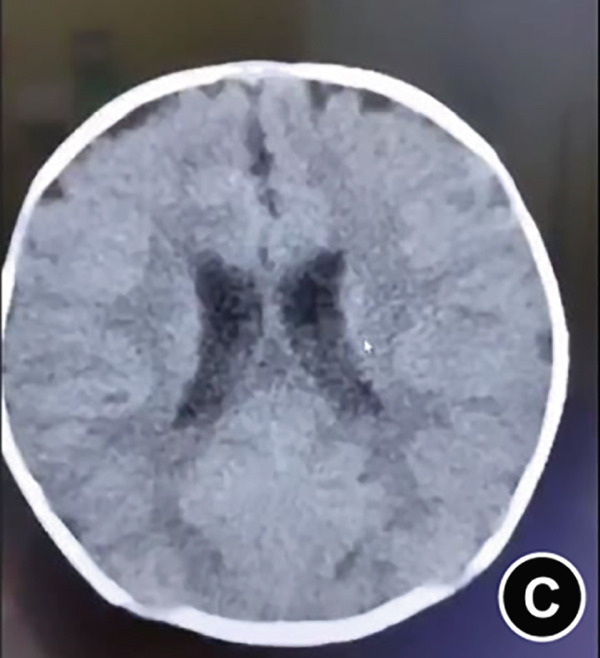
(c)

**Figure:**
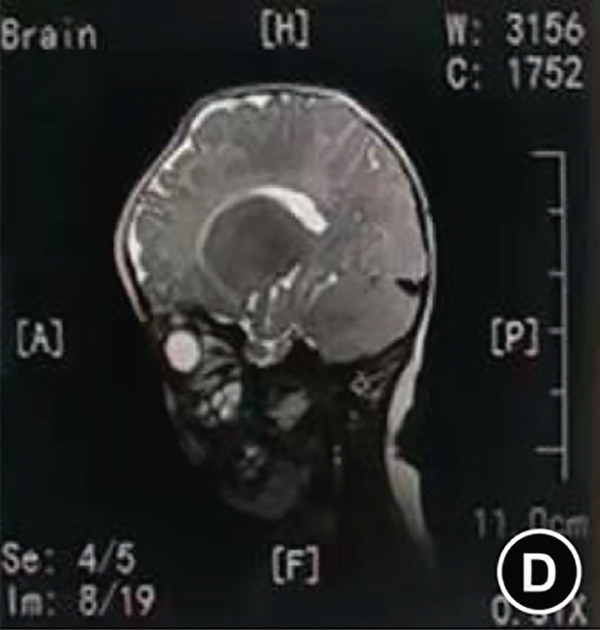
(d)

**Figure:**
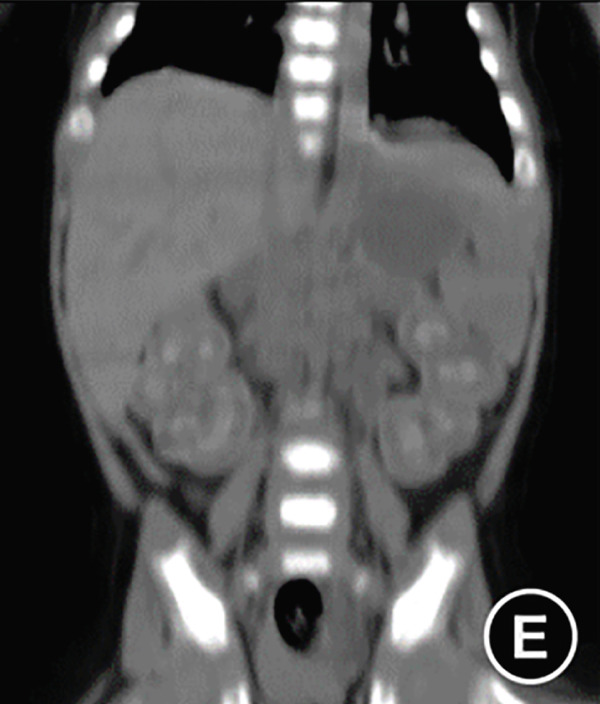
(e)

**Figure:**
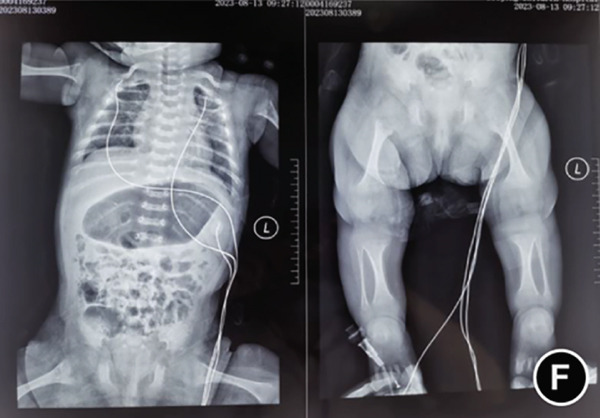
(f)

**Figure:**
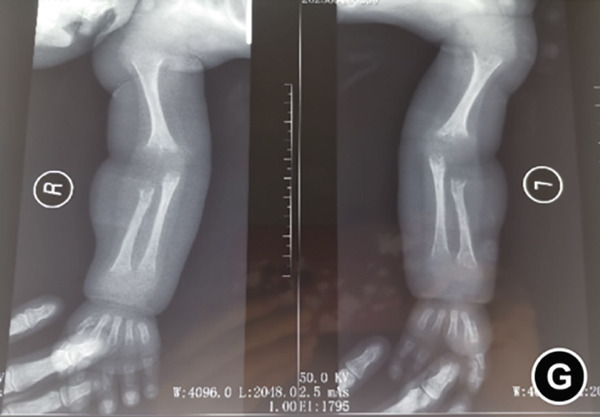
(g)

### 3.3. Identification of the Ovality and Parentage of the Twins

Patients II‐2 and II‐3 were born with only one placenta and were observed to have monochorionic double amniotic sacs on early pregnancy ultrasound and at birth. Based on the WES data of the five family members, a pairwise comparison was carried out using KING2.3.1 software, and kinship coefficients were generated to determine the parentage of the twins. II‐2 and II‐3 had kinship coefficients of 0.4879 and were determined to be monozygotic twins (Table S1).

### 3.4. Gene Sequencing Results

**Figure 2:**
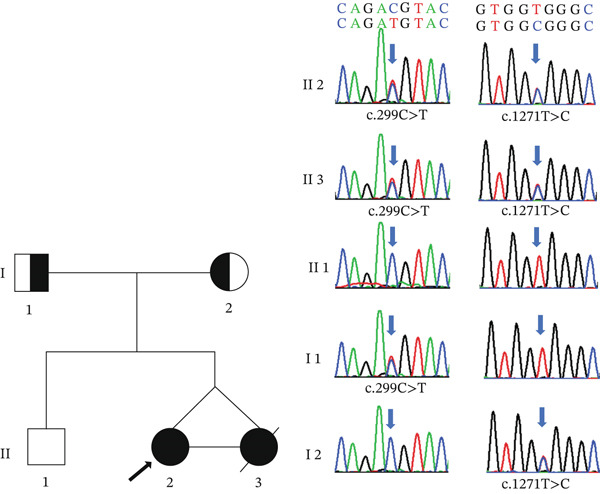
Pedigree map of two monozygotic twin female infants with HPP and results of Sanger sequencing of the *ALPL* gene. Older sister II‐2 (proband) and younger sister II‐3 were monozygotic twin female infants with HPP. II‐2 survived to the last follow‐up at age 15 months. II‐3 died of a pulmonary infection at the age of 11 months. 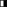 indicates a single heterozygous mutation in the *ALPL* gene c.299C>T from the father I‐1 (37 years, healthy). 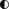 indicates a single heterozygous mutation in the *ALPL* gene c.1271T>C from the mother I‐2 (34 years, healthy). 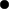 indicates compound heterozygous mutations in the *ALPL* genes c.299C>T and c.1271T>C. 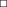 indicates the wild‐type *ALPL* gene. The older brother II‐1 (12 years, healthy) does not carry any *ALPL* gene mutation. HPP, hypophosphatasia.

No pathogenic CNVs, heterozygous deletions, or uniparental diploidies were detected with CNV testing. WES revealed the presence of compound heterozygous mutations in the *ALPL* genes of II‐2 and II‐3, and primers (Table S2) were designed and verified using Sanger sequencing (Figure 2). Genotypes of II‐2 and II‐3 contained Exon 5 c.299C>T (p.Thr100Met) and exon 11 c.1271T>C (p.Val424Ala), respectively. The father was a single heterozygous mutation carrier for c.299C>T (p.Thr100Met), the mother was a single heterozygous mutation carrier for c.1271T>C (p.Val424Ala), and the older brother (c.299C and c.1271T) carried the wild‐type alleles. The *ALPL* c.299C>T (p.Thr100Met) variant results in a substitution of methionine for threonine at residue 100, which was deemed pathogenic based on ACMG criteria (PM2_Supporting, PM3_Strong, and PP3_Strong) [18]. In silico analysis using SIFT, PolyPhen‐2, MutationTaster, and GERP++ unanimously predicted the c.299C>T (p.Thr100Met) variant to be harmful. The *ALPL* c.1271T>C (p.Val424Ala) variant resulted in a change at residue 1424 from valine to alanine. This substitution was classified as likely pathogenic based on ACMG criteria (PM2_Supporting, PM3_Strong, PM5, and PP3) [18]. Computational predictions were inconsistent: SIFT predicted a benign effect, whereas PolyPhen‐2, MutationTaster, and GERP++ indicated a harmful outcome.

### 3.5. Clinical Forms and Functional Analysis of the *ALPL* Gene

**Figure 3:**
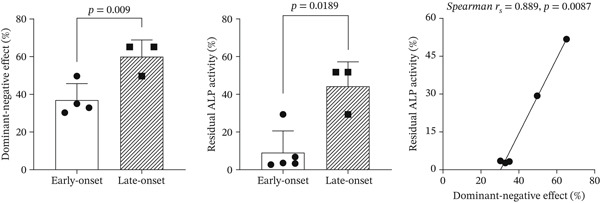
Comparison of differences and correlation analysis for residual ALP activity and dominant‐negative effect of genotype in relation to c.299C>T *ALPL* mutations. ALP, alkaline phosphatase.

Data on genotype, phenotype, mutant residual ALP activity (percentage), and dominant‐negative effects (percentage) for c.299C>T, c.1270G>A, and c.1271T>C were obtained from the *ALPL* Gene Variant Database (Table 2). As of February 2026, the database contained 498 nucleotide variants and over 1000 genotypes. Of these, six entries involved the c.299C>T (p.Thr100Met) variant. A single heterozygous mutation was identified in an asymptomatic carrier exhibiting the biochemical phenotype, as well as in a childhood and an adult HPP case. One homozygous mutation was documented in a case of perinatal HPP. The remaining four cases involved compound heterozygous mutations: [c.299C>T (p.Thr100Met); c.571G>A (p.Glu191Lys)] in an infantile and an adult HPP case and [c.299C>T (p.Thr100Met); c.227A>G (p.Gln76Arg)], [c.299C>T (p.Thr100Met); c.982T>C (p.Phe328Leu)], and [c.299C>T (p.Thr100Met); c.1258G>A (p.Gly420Ser)], each linked to perinatal HPP. Due to the low prevalence of genetic diseases, including HPP, as well as the limited number of cases and genotypes available in this study, *ALPL* c.299C>T genotypes were categorized as early‐onset (< 6 months) and late‐onset (≥ 6 months). Two independent sample *t*‐tests and Spearman′s correlation analyses were performed. Early‐onset data were approximately normally distributed and were analyzed using a *t*‐test. The results showed statistically significant differences in the dominant‐negative effect and mutant residual ALP activity between early‐ and late‐onset types of c.299C>T‐associated *ALPL* mutations (*p* = 0.009 and 0.0189, respectively; Figure 3). Moreover, Spearman′s correlation analysis of the linear relationship between the dominant‐negative effect and mutant residual ALP activity showed a highly positive correlation coefficient (*r*_*s*_ = 0.889, *p* = 0.0087; Figure 3).

**Table 2:** Genotypes and phenotypes of hypophosphatasia in the *ALPL* Gene Variant Database.

Location	Base change	Amino acid change	Pathogenicity	Phenotype	Genotype		Mutant residual ALP activity (%)		Dominant‐negative effect (%)	
					Allele 1	Allele 2	Allele 1	Allele 2	Allele 1	Allele 2
Exon 5	c.299C>T	p.Thr100Met	Pathogenic	Asymptomatic with biochemical phenotype	c.299C>T	WT	3.55	100	30.3	100
				Childhood	c.299C>T	WT	3.55	100	30.3	100
				Adult	c.299C>T	WT	3.55	100	30.3	100
				Adult	c.299C>T	c.571G>A	3.55	55.13	30.3	69.1
				Infantile	c.299C>T	c.571G>A	3.55	55.13	30.3	69.1
				Perinatal	c.299C>T	c.1258G>A	3.55	1.85	30.3	35.5
				Perinatal	c.299C>T	c.227A>G	3.55	3.0	30.3	39.8
				Perinatal	c.299C>T	c.299C>T	3.55	3.55	30.3	30.3
				Perinatal	c.299C>T	c.982T>C	3.55	10	30.3	ND

Exon 11	c.1271T>C	p.Val424Ala	Pathogenic	Adult	c.1271T>C	c.1271T>C	ND	ND	ND	ND

Exon 11	c.1270G>A	p.Val424Met	Pathogenic	Adult	c.1270G>A	c.1270G>A	58.5	58.5	88.4	88.4

*Note:* Mutations associated with the c.299C>T, c.1271T>C, and c.1270G>A variants in the *ALPL* gene are listed. Data are available from the *ALPL* Gene Variant Database (https://alplmutationdatabase.jku.at/) at the Johannes Kepler University Linz (JKU), Austria [20]. Mutant residual ALP activity (percentage): < 25% suggests low residual ALP activity, and > 50% suggests high residual ALP activity [21]. Dominant‐negative effects (percentage): In vitro experiments detected the ratio of heterozygous mutant ALP activity to wild‐type activity, with < 40% indicative of the presence of dominant‐negative [21].

Abbreviations: ALP, alkaline phosphatase; ND, no data; WT, wild type.

### 3.6. Protein 3D Structure

**Figure 4:**
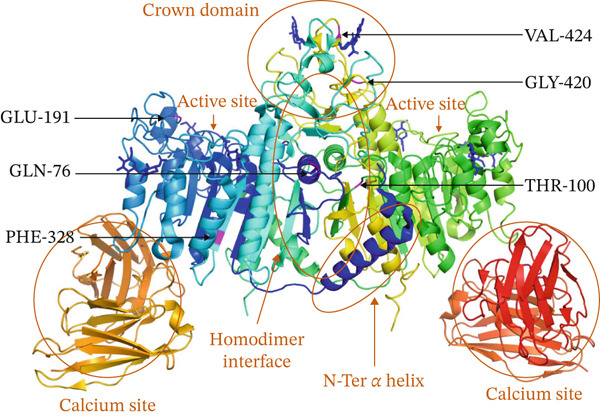
Localization map of *ALPL* gene mutants in the 3D structure of TNSALP protein. *ALPL* gene mutations associated with the c.299C>T variant include c.571G>A (p.Glu191Lys), c.982T>C (p.Phe328Leu), c.227A>G (p.Gln76Arg), c.299C>T (p.Thr100Met), and c.1258G>A (p.Gly420Ser). *ALPL* gene mutations associated with the c.1271T>C variant include c.1270G>A (p.Val424Met) and c.1271T>C (p.Val424Ala). Five functional domains comprise the active site, homodimer interface, crown domain, N‐terminal *α*‐helix, and calcium‐binding site. Magenta‐colored bars indicate positions in relation to *ALPL* mutations (c.299C>T, c.1270G>A, and c.1271T>C). N‐Ter, N‐terminal; TNSALP, tissue‐nonspecific alkaline phosphatase.

Referring to the cryoelectron microscopy structure of the TNSALP protein (PDBID_7YIX) [22], *ALPL* gene mutations (Table 2) were localized in different functional domains. c.571G>A (p.Glu191Lys) and c.982T>C (p.Phe328Leu) were located in the active site. c.227A>G (p.Gln76Arg) and c.299C>T (p.Thr100Met) were located at the homodimer interface. c.1258G>A (p.Gly420Ser), c.1270G>A (p.Val424Met), and c.1271T>C (p.Val424Ala) were located in the crown domain (Figure 4). The mutations (Table 2) were not located in the N‐terminal *α*‐helix region or calcium‐binding site.

## 4. Discussion

This study provided new insights into the atypical manifestations of infantile HPP in monozygotic twins. The twins presented with bulging fontanel at the age of 3 months simultaneously; both survived the first 6 months of infancy, and one of them lived for over 15 months. A pathogenic compound heterozygous mutation in the *ALPL* gene—comprising one severe and one mild symptomatic allele—resulted in an intermediate phenotype between perinatal and adult HPP.

Infantile HPP is associated with unfavorable outcomes. The earlier the onset of HPP, the more severe the symptoms and the worse the prognosis. Fifty percent of patients with infantile HPP die within the first year of life due to severe rickets with thoracic deformities, leading to pulmonary insufficiency and recurrent pneumonia or vitamin B_6_–responsive seizures [2, 3, 11]. Only a few children with infantile HPP survive to the age of 5 years, with slow weight gain, hypercalcemia with renal calcium deposition, and severe rickets in the early course of the disease; they subsequently present with short stature, premature tooth loss, and premature closure of cranial sutures [3, 11]. The identical twins (II‐2 and II‐3) in this study were symptomatic at 3 months of age. They were hospitalized at the age of 6 months when they were found to have insufficient ossification of the long bones of the extremities without severe thoracic deformities. Unlike most patients with severe infantile HPP presenting with hyperphosphatemia [2, 6], the twins had normal serum phosphorus levels. Although PLP levels were elevated, no convulsions were observed. This suggests that the children′s ALP hydrolytic activity was partly retained on the surface of the neuronal cell membranes, where it could transport pyridoxal intracellularly and play a role in vitamin B_6_ metabolism. Patients II‐2 and II‐3 were hospitalized for their first episode of pneumonia at 7 months of age and were discharged without respiratory support. II‐3 died from a second episode of pneumonia at 11 months of age. II‐2 survived for more than 1 year. These two twins had similar postnatal clinical symptoms and biochemical changes and survived for a long time, indicating that mutations in the *ALPL* gene did not fatally affect TNSALP activity.

A novel compound heterozygous mutation in the *ALPL* gene was identified in the affected children, comprising variants in Exon 5 (c.299C>T; p.Thr100Met) and Exon 11 (c.1271T>C; p.Val424Ala). This genotype was not listed in the *ALPL* Gene Variant Database (Table 2) [20]. To date, six cases of c.299C>T (p.Thr100Met)‐related genotypes have been reported, whereas only one homozygous case of c.1271T>C has been documented in an adult with HPP from China [23]. Referring to the 3D structural map of the TNSALP protein (Figure 4), *ALPL* gene mutations in perinatal and infantile HPP were primarily spread in the activation site and the homodimer interface region, and these mutations in adult HPP were mainly distributed in the crown domain. Most of these domains were associated with severe *ALPL* mutations. Further experiments confirmed that *ALPL* mutations with dominant‐negative effects were mainly distributed in the important structures of the active site, crown domain, and homodimer interface [5, 21].

A severe mutation in the *ALPL* gene largely has a dominant‐negative effect, with defective residual ALP activity. In the c.299C>T *ALPL* mutation, the residual ALP activity of mutants remained at only 3.55%, correlating with a low level of activity, and the value of the dominant‐negative effect was 30.3% (i.e., less than 40%), suggesting the presence of a dominant‐negative effect [21]. Analysis of the c.299C>T‐related genotypes and phenotypes (Table 2 and Figure 4) revealed that the clinical phenotype of HPP was associated with dominant‐negative effects and mutant residual ALP activity. Notable decreases were observed in the dominant‐negative effect alteration, and the allelic mutant residual ALP activity decreased when early‐onset HPP was compared with late‐onset HPP. Spearman′s correlation analysis indicated that dominant‐negative effects and mutant activity were highly positively correlated. Biallelic *ALPL* mutations of severe loss‐of‐function genotypes result in decreased mutant activity and a significant dominant‐negative effect. Furthermore, compound heterozygous mutations were associated with an early onset of severe clinical phenotypes in perinatal and infantile HPP. Contrastingly, compound heterozygous mutations in mild symptomatic genotypes can lead to childhood or adult HPP [10].

Functional testing of mutant activity or a dominant‐negative effect of the allele c.1271T>C (p.Val424Ala) has not been reported in the *ALPL* Gene Variant Database (Table 2). However, a homozygous mutation in the *ALPL* gene c.1270G>A (p.Val424Met) in adult HPP has been reported from a laboratory at the University of Versailles‐Saint Quentin en Yvelines (France) [24]. c.1270G>A (p.Val424Met) is similar to the genotype of the mutation c.1271T>C (p.Val424Ala) in this study, which occurs at amino acid residue 424 of TNSALP and is located in the crown domain (Table 2 and Figure 4). Structurally, methionine, with its longer, unbranched thioether‐containing side chain (molar mass 149.2 g/mol), is considerably bulkier than valine (isopropyl group, molar mass 117.1 g/mol) or alanine (single methyl group, molar mass 89.1 g/mol). This increased bulk and potential for steric hindrance in the crown domain (amino acid residue 424, Figure 4) could make p.Val424Met more disruptive to protein structure or interactions with ligands than p.Val424Ala. Functionally, p.Val424Met shows higher residual ALP activity (58.5%) and no dominant‐negative effect (88.4%) [21]. Furthermore, c.1271T>C (p.Val424Ala) is predicted to be likely pathogenic by ACMG/AMP [18], ClinVar, and Mastermind [20]. In contrast, c.1270G>A (p.Val424Met) is consistently predicted to be pathogenic by ACMG/AMP [18] and Mastermind [20]. Clinically, c.1270G>A (p.Val424Met) has been reported in a homozygous case of adult HPP, but not in severe perinatal or infantile forms [23]. Therefore, we propose that the pathogenicity of c.1271T>C (p.Val424Ala) is likely lower than that of c.1270G>A (p.Val424Met). Given that the homozygous mutation c.299C>T (p.Thr100Met) is associated with perinatal HPP, it can be hypothesized that the compound heterozygous mutations in the *ALPL* gene [c.299C>T (p.Thr100Met) and c.1271T>C (p.Val424Ala)] in the affected twins were combinations of the severe and mild genotypes in *ALPL* mutations, and the severity of the phenotype may be intermediate between that of perinatal and adult HPP [10].

As asymptomatic *ALPL* carriers, the parents each harbored a heterozygous mutation—c.299C>T (p.Thr100Met) in the father and c.1271T>C (p.Val424Ala) in the mother—and both exhibited biochemical phenotype (low ALP and elevated PLP). In contrast, their elder brother displayed a wild‐type *ALPL* genotype. Single heterozygous mutations exhibit variable expressivity: c.299C>T (p.Thr100Met) has been associated with childhood HPP, adult HPP, and asymptomatic carriers with a biochemical phenotype, consistent with a dominant‐negative effect (Table 2). This variability aligns with a previous study estimating incomplete penetrance at 32% for autosomal dominant HPP [9], thereby accounting for the father′s healthy clinical status despite a single heterozygous mutation. Additionally, perinatal benign HPP needs to be differentiated because the twins in this study had the disease onset in early infancy. As patients with perinatal benign HPP survive to birth, 85% of affected infants carry a pathogenic and severe *ALPL* genotype from their mothers, whose fetal skeletal development is severely affected in utero [25]. Based on the functional analysis, c.1271T>C (p.Val424Ala) from the mother of the twins was considered a mild genotype. Fetal ultrasound did not suggest shorter or curved changes in the long bones; therefore, the twins were not considered to have perinatal benign HPP.

Monozygotic twin female infants (II‐2 and II‐3) had normal fontanels at birth, with simultaneous bulging at 3 months of age, large eyes, and low‐density shadows under the fontanels detected by cranial CT at 6 months of age to exclude intracranial occupations and infections. The head circumference of II‐2 and II‐3 at birth was in the 3rd–25th percentile, corresponding to their age, and was smaller than that in the 3rd percentile at age 6 months. II‐2′s head circumference at age 15 months was smaller than that in the 3rd percentile. These results suggest that the head circumference of these twins was smaller than normal during infancy brain development. While the cerebrospinal fluid pressure was nearly normal, the bulging fontanel may have helped alleviate the increased intracranial pressure. Fraser [2] first reported that some typical Canadian infants with infantile HPP had bulging fontanel, large and obvious eyeballs, and abnormal cranial ossification, leading to slow growth of the head circumference, which was lower than the 15th or even 3rd percentile for their age. Bulging fontanel may be due to the inconsistency between the growth rate of the head circumference and the increase in brain volume during early infancy. Premature closure of cranial sutures affects the skull significantly during infancy and early childhood, and the potential risk of increased intracranial pressure is alleviated until adolescence [6]. Additionally, idiopathic intracranial hypertension was reported in two cases of infantile HPP, with bulging fontanel, cerebrospinal fluid pressure of 15–77 cmH_2_O, mild enlargement of the third and lateral ventricles on partial craniocerebral CT, and no abnormalities in fundus and neurologic examination. The cerebrospinal fluid pressure decreased to normal after treatment with acetazolamide, mannitol, and corticosteroid [26, 27]. These findings were associated with abnormal calcium and phosphorus metabolism, which affected cellular energy conversion, cell membrane structural function, and intercellular ion concentration [26].

This study has some limitations. First, due to financial constraints in the family, the parents did not agree with the administration of asfotase alfa to the affected twins for TNSALP enzyme replacement therapy. More indicators from focused examinations or regular follow‐ups after the first hospitalization were unavailable. Second, the residual ALP activity and dominant‐negative effect of the *ALPL* gene c.1271T>C (p.Val424Ala) mutant require functional characterization. Del Angel et al. [21] performed transient cellular transfection of 155 variants in *ALPL* to test the residual activity of TNSALP. Only 90 variants were shown to decrease mutant residual ALP activity, and 24 variants had dominant‐negative effects. In vitro assays cannot simulate complete intracellular life processes or mimic in vivo nongenetic influences [12]. We averaged the in vitro values for both alleles in each c.299C>T‐related heterozygous genotype. Whether these averages correspond to actual serum ALP levels warrants further investigation.

## 5. Conclusions

In summary, a novel pathogenic biallelic heterozygous *ALPL* mutation was identified in the female monozygotic twins with infantile HPP. The twins had nearly identical genotypes that were similar in clinical features and biochemical abnormalities. These findings were further supported by an analysis of the JKU database, which demonstrated that alterations in mutant residual ALP activity and dominant‐negative effects could influence HPP severity.

## Author Contributions

All authors contributed to the conception and design of this study. Material preparation, data collection, and analyses were performed by Luna Hao, Na Huang, Hui Li, Xiaoyun Li, and Zekun Hao. Luna Hao, Yilun Tao, Juyu Zhuang, and Feng Zhao drafted the manuscript. Luna Hao and Feng Zhao are co‐corresponding authors.

## Funding

This study was funded by the Fujian Provincial Natural Science Foundation of China (2022J01436).

## Disclosure

All the authors have read and approved the final version of the manuscript.

## Ethics Statement

This study was performed in line with the principles of the Declaration of Helsinki, and it was approved by the Medical Ethics Committee of Changzhi Maternal and Child Health Hospital (Shanxi, China) (Approval No. CZSFYLL2024‐006). Written informed consent was obtained for all participants (for minors, guardians signed the consent forms).

## Conflicts of Interest

The authors declare no conflicts of interest.

## Supporting information


**Supporting Information** Additional supporting information can be found online in the Supporting Information section. Table S1: Pairwise kinship coefficients in the family of HPP monozygotic female twins. Table S2: Sequencing of upstream and downstream primers for Exons 5 and 10 of *ALPL* and amplification conditions.

## Data Availability

The data that supports the findings of this study are available in the supplementary material of this article.
