# Supplementary material for: ALPL Mutations With Dominant‐Negative Effect in Infantile Hypophosphatasia Monozygotic Twins
Source: Hum Mutat. 2026 Apr 15;2026:9913394. doi: 10.1155/humu/9913394 (PMC13080685; doi:10.1155/humu/9913394)
Supplement: Supplementary file 1 — Supporting Information Additional supporting information can be found online in the Supporting Information section. Table S1: Pairwise kinship coefficients in the family of HPP monozygotic female twins. Table S2: Sequencing of upstream and downstream primers for Exons 5 and 10 of ALPL and amplification conditions. [file HUMU-2026-9913394-s001.docx]

**Supplementary Table 1. A list of pair-wise kinship coefficients in the family of monozygotic female twins with HPP**

| No. | Paired-relationship | Kinship coefficient | Degree |
| --- | --- | --- | --- |
| 1 | II-3, I-1 | 0.2725 | 1st-degree |
| 2 | II-3, I-2 | 0.2825 | 1st-degree |
| 3 | I-1, I-2 | 0.0678 | unrelated degree |
| 4 | II-1, II-3 | 0.2662 | 1st-degree |
| 5 | II-2, II-3 | 0.4879 | duplicate/monozygotic twin |
| 6 | I-1, II-1 | 0.2779 | 1st-degree |
| 7 | I-1, II-2 | 0.2716 | 1st-degree |
| 8 | I-2, II-1 | 0.2803 | 1st-degree |
| 9 | I-2, II-2 | 0.283 | 1st-degree |
| 10 | II-1, II-2 | 0.2676 | 1st-degree |

Note: an estimated pair-wise kinship coefficient range >0.354, [0.177, 0.354], [0.0884, 0.177] and [0.0442, 0.0884] corresponds to duplicate/monozygotic twin, 1st-degree, 2nd-degree, and 3rd-degree relationships respectively (<https://www.kingrelatedness.com/manual.shtml>). HPP, hypophosphatasia.

**Supplementary Table 2. Sequencing of upstream and downstream primers of exon 5 and exon 10 of *ALPL* gene and amplification conditions**

| Primer | Base sequence | Annealing temperature (℃) | Amplified product size (bp) |
| --- | --- | --- | --- |
| *ALPL*-299F | GGCTTCAGTGGGCAGTGG | 55 | 403 |
| *ALPL*-299R | CCTTCCCATGACACAGTCAGA |  |  |
| *ALPL*-1271F | CCCTTCAAAGAAGATCCCAGG | 55 | 323 |
| *ALPL*-1271R | TGACCTTGACCACAAGCTGAC |  |  |

Note: NCBI GenBank *ALPL* Gene mRNA Accession Version NM_000478.6
